# Distinct Skin Microbiome and Skin Physiological Functions Between Bedridden Older Patients and Healthy People: A Single-Center Study in Japan

**DOI:** 10.3389/fmed.2020.00101

**Published:** 2020-04-08

**Authors:** Satoshi Nagase, Kazuhiro Ogai, Tamae Urai, Kana Shibata, Emi Matsubara, Kanae Mukai, Miki Matsue, Yumiko Mori, Miku Aoki, Defa Arisandi, Junko Sugama, Shigefumi Okamoto

**Affiliations:** ^1^Department of Clinical Laboratory Science, Faculty of Health Sciences, Institute of Medical, Pharmaceutical and Health Sciences, Kanazawa University, Kanazawa, Japan; ^2^Wellness Promotion Science Center, Institute of Medical, Pharmaceutical and Health Sciences, Kanazawa University, Kanazawa, Japan; ^3^Advanced Health Care Science Research Unit, Innovative Integrated Bio-Research Core, Institute for Frontier Science Initiative, Kanazawa University, Kanazawa, Japan; ^4^Department of Clinical Nursing, Faculty of Health Sciences, Institute of Medical, Pharmaceutical and Health Sciences, Kanazawa University, Kanazawa, Japan

**Keywords:** microbiome, skin, skin physiological function, pressure injury, wound

## Abstract

With the increase in the older populations, the number of bedridden older patients is becoming a matter of concern. Skin microbiome and skin physiological functions are known to change according to lifestyle and community; however, such changes in case of movement- and cleaning-restricted bedridden older patients have not yet been revealed. To address this issue, we analyzed skin microbiome and skin physiological functions, including pH, hydration, sebum level, and transepidermal water loss (TEWL), of bedridden older patients, compared with those of ambulatory older and young individuals. For this analysis, we enrolled 19 healthy young and 18 ambulatory older individuals from the community and 31 bedridden older patients from a single, long-term care hospital in Japan. The area of interest was set to the sacral (lower back) skin, where pressure injuries (PIs) and subsequent infection frequently occurs in bedridden older patients. We observed a higher number of gut-related bacteria, fewer commensals, higher skin pH, and lower TEWL on the sacral skin of bedridden older patients than on that of young or ambulatory older individuals. In addition, we observed that 4 of the 31 bedridden older patients developed PIs during the research period; a higher abundance of pathogenic skin bacteria were also observed inside the PI wounds. These findings imply distinct skin microbiome and skin physiological functions in bedridden older patients in comparison with healthy individuals and may suggest the need for more stringent cleaning of the skin of bedridden older patients in light of the closeness of skin and wound microbiome.

## Introduction

Skin serves as a protectant against external insult (e.g., pressure, shock, ultraviolet light, chemicals, and pathogens), as well as retaining the fluid within the body. It is now widely recognized that the skin barrier consists not only of the “wall” of skin cells but also of various bacteria (1). For example, the major skin commensal *Cutibacterium* (formerly *Propionibacterium*) *acnes* is known to produce fatty acids, which in turn keep skin pH mildly acidic and prevent colonization of transient bacteria (2). *Staphylococcus epidermidis* is a major source of glycerol on the skin, which is responsible for skin water retention (3). Depletion of *S. epidermidis* can cause skin inflammation (4), and several *Staphylococcus* spp. can produce antimicrobial peptides to prevent colonization of pathogens (5). Therefore, dysbiosis of the skin microbiome can lead to the deterioration of skin physiological and barrier functions.

Skin microbiome has been reported to change according to various factors, such as lifestyle, age, and community (6). Chronological aging itself can influence the skin microbiome; for example, the abundance of *Cutibacterium* spp. has been found to be lower in older individuals (7, 8). Interestingly, one study showed that the skin microbiomes of older and ambulatory residents in nursing homes were very different from those of age-matched community participants (9). This study implies that there is a synergetic effect between aging and dwelling environment, and the study proposed skin microbiome as a potential source of drug-resistant bacteria in hospitals. Because the situation of bedridden older patients can be considered more severe (in terms of severely reduced daily activities such as moving, eating, and bathing) than that of ambulatory older individuals, it is possible that the skin microbiome of bedridden older patients might show yet another dysbiotic pattern, compared with ambulatory individuals. Given the increasing population of bedridden older patients and their susceptibility to infectious diseases, it is important to be able to identify dysbiotic conditions in bedridden older patients.

Skin microbiomes can be altered via changes in skin physiological functions and vice versa. For example, several commensal bacteria on the skin can maintain a slightly acidic pH, and such an acidic condition can in turn facilitate the growth of these commensal bacteria (10); pathogenic bacteria can flourish under high skin pH (11); and the levels of facial skin hydration (moisture) and sebum are related to different microbial patterns (12, 13). Because bedridden older patients usually use diapers and their skin is in contact with urine and/or feces for a long time, incontinence-associated skin deterioration, such as increase in skin pH, and dysbiosis can also be a problem (14, 15). Therefore, to understand the nature of the skin of a bedridden older patient, it is necessary to identify the differences in both skin physiological functions and skin dysbiosis.

Another problem among bedridden older patients is the occurrence of pressure injuries (PIs). A PI is defined as “localized damage to the skin and underlying soft tissue over a bony prominence or related to a medical or other device” (16); therefore, bedridden older patients with low mobility are more likely to develop PIs (17). The worse part of PIs is that, once PIs become infected, the wounds can lead to life-threatening osteomyelitis and sepsis (18). Because the bacteria inside the wound are reportedly derived from the surrounding skin (19, 20), we hypothesized that skin dysbiosis could be a source of the pathogenic bacteria inside the wound after PI onset.

The aim of this study, therefore, was to compare the skin microbiome and skin physiological functions of bedridden older patients with those of ambulatory older people and young individuals. We further followed up the patients for 2 months to observe the onset of PIs and to reveal the relationship between skin and wound microbiome.

## Materials and Methods

### Ethical Considerations

This study was approved by the Medical Ethics Committee of Kanazawa University (approval nos. 632 and 765) and was conducted in accordance with the Declaration of Helsinki and the Microorganism Safety Management Regulations of Kanazawa University. Before the collection of skin microorganisms, all the participants or their family received an explanation for the research and provided written informed consent. All the collected samples were processed in a biosafety level-2 laboratory.

### Study Design, Participants, and Settings

This was a prospective cohort study. We recruited 19 healthy young (20–29 years old; 12 men and 7 women), 18 ambulatory older individuals (66–95 years old; 7 men and 11 women), and 31 bedridden older (71–100 years old; 9 men and 22 women) patients. The healthy young and ambulatory older participants were recruited via the network of the researchers. The bedridden older patients were recruited in one long-term care hospital in Ishikawa prefecture, Japan, through the referral of the nurses or physicians in charge of the ward.

We excluded participants who had skin disorders, including atopic dermatitis, psoriasis, and PIs, on the target region.

Target regions were set as the sacral region of the skin, where PIs are reported to occur frequently in bedridden patients (21–23), and upper back skin as a reference (Supplementary Figure 1).

At the time of inclusion, the bedridden patient's demographic information, peak interface pressure, skin physiological functions, and skin bacteria of the target region were collected. The patients were followed up every week for up to 2 months to check if they had a new PI. We used the DESIGN-R scoring system (24) to assess the onset and depth of PIs. If a new PI was found, the bacteria on (depth = d1; non-blanchable erythema) or inside (depth ≥ d2; wound having epidermal loss) the wound were collected. Two months after recruitment, the skin microbiome was collected, and the skin physiological functions were analyzed. The whole research period was between October 2015 and November 2017, including all the participants.

### Participants' Information

Participants' demographic and medical information, including age, sex, gastric fistula, underlying diseases, and medication, was collected through interview or from the medical record. Braden Scale scores (25) were assessed by trained nurses.

### Skin Physiological Functions

Derma Unit SSC3 (Courage + Khazaka electronic GmbH, Inc., Cologne, Germany) was used to measure skin hydration, sebum level, and pH. Transepidermal water loss (TEWL) was measured using a VapoMeter® (Delfin Technologies, Ltd, Kuopio, Finland). All the measurements were performed three times, and the average was calculated. A Palm-Q (CR-490; CAPE Co., Ltd., Kanagawa, Japan) was used to measure the peak interface pressure of the sacral region.

### Collection of Skin Bacteria

Skin bacteria were collected by the tape-stripping method based on the previous study (26). First, the skin of the target region was gently cleansed by a trained nurse with a sterile normal saline solution (Otsuka Pharmaceutical Co., Ltd, Tokyo, Japan) to eliminate any transient contamination. After cleansing and drying the skin, an air-permeable medical tape (4.0 × 5.0 cm) was attached to the skin with silicone glue for 1 min. Then, the tape was removed with sterile tweezers and placed in a sterile container. When the PI had epidermal loss (i.e., depth ≥ d2), the wound site bacteria were collected with a flocked swab (HydraFlock; Puritan Medical Products, LLC, ME, United States) presoaked with sterile normal saline. All the samples were stored at −80°C until DNA extraction.

### DNA Extraction

The whole DNA was extracted from the collected tapes and swabs based on the previous study (26). In brief, DNA was extract by means of a QIAamp DNA Mini kit (QIAGEN N.V., Venlo, Netherlands) in accordance with the appendix protocol “Isolation of genomic DNA from Gram-positive bacteria.” The collected tape or swab head was treated with a lysozyme–lysostaphin enzyme solution [20 mg/ml lysozyme (FUJIFILM Wako Pure Chemical Corp., Osaka, Inc., Japan) and 0.2 mg/ml lysostaphin (FUJIFILM Wako Pure Chemical Corp.) in 20 mM Tris–HCl (pH 8.0), 2 mM EDTA, and 1.2% Triton-X 100] at 37°C for 30 min, followed by subsequent processing according to the manufacturer's instructions.

### Next Generation Sequencing (NGS)

The extracted DNA samples were processed for 16S ribosomal RNA (rRNA) gene sequencing by the next generation sequencing (NGS) method (26). The hypervariable regions 3–4 (V3–V4) of the 16S rRNA gene was amplified with *Ex Taq*® Hot Start Version (TaKaRa Bio Inc., Shiga, Japan) and TaKaRa PCR Thermal Cycler Dice® Gradient (TaKaRa Bio Inc.). We chose the V3–V4 region for the target of amplification in this study according to the findings by Castelino et al. and Zeeuwen et al. who reported the advantage of using the V3–V4 region for skin microbiome studies (27, 28). The PCR fragments were purified by Agencourt AMPure XP magnetic beads (Beckman Coulter, Inc., CA, United States). After index PCR and purification, the concentration of indexed fragments was measured with Qubit® dsDNA HS Assay Kit using Qubit® 3.0 (Thermo Fisher Scientific, Inc., MA, United States). The equimolar mixture of the products was sent to FASMAC Co., Ltd (Kanagawa, Japan) for Illumina MiSeq sequencing. All the sequence data were deposited in the DNA Data Bank of Japan (DDBJ; accession number: DRA007616).

### Microbiome Analysis

Microbiome analysis was performed as described elsewhere (26) with slight modifications. The raw pair-end sequences were filtered by Sickle (version 1.33) (29) and combined by PANDAseq (version 2.11) (30). The chimeric sequences were removed using the USEARCH (version 10.0.240_i86linux32) (31) and Silva 16S rRNA database (release 132; 97_otus.fasta) (32). Nonchimeric sequences were filtered by size (>300 bp).

The operational taxonomic unit (OTU) selection, with 97% similarity threshold from the nonchimeric sequences, was performed using the “pick_de_novo_otus.py” command in Qiime (version 1.9.1) (33), using the Silva 16S rRNA gene database (release 132) as a taxonomy database. Finally, the global singletons were excluded using the “filter_otus_from_otu_table.py” command in Qiime. For alpha diversity analysis, all sequences were rarefied at 13,783 depth (minimum read number among all samples). After rarefaction, alpha diversity indices [phylogenetic diversity (PD_whole_tree), observed OTUs, Chao1, and Shannon index] were calculated. For beta diversity analysis, weighted and unweighted UniFrac distances were calculated, followed by visualization by principal coordinate analysis (PCoA).

### Statistics

R Statistical Package (version 3.5.1) was used to perform all the statistical analyses (34). Box plots represent the first quartile, median, and third quartile, with first quartile + 1.5 × interquartile range (IQR), 3rd quartile−1.5 × IQR whiskers and outliers as points. Participants' characteristics and relative abundance among each group were compared using the Mann–Whitney *U-*test, Kruskal–Wallis test followed by Steel–Dwass *post-hoc* test using “pSDCFlig” command in the “NSM3” package (35), or Fisher's exact test. *P*-values were adjusted by the Benjamini–Hochberg's false-discovery rate control. A heat map of Box-Cox transformed abundance of microbial taxa was made using the “heatmap.2” command in the “gplots” package (36) of R, along with the Manhattan distance and unweighted pair group method with arithmetic mean-based dendrograms (37). Canonical correspondence analysis (CCA) was performed to determine the effects of skin physiological functions to the skin microbiome using the “cca” command in “vegan” package (38) of R. Similarity of the bacterial composition between each participant group was assessed by permutational multivariate analysis of variance (PERMANOVA) using the “adonis” command in the “vegan” package (10,000 simulations) of R. Correlation of the relative abundance between two groups was assessed by the Spearman's rank correlation coefficient, denoted herein as ρ.

## Results

### Participants' Information

Table 1 summarizes participants' information. Regarding the age of the participants, the Kruskal–Wallis test showed significant difference among groups (*P* < 0.01), whereas no significant difference was found between ambulant older people and bedridden older patient groups (*P* = 0.06; Steel–Dwass test). Some bedridden patients had cerebrovascular disease (18 out of 31), congestive heart failure (6/31), diabetes mellitus (3/31), and urinary tract infectious disease (3/31) as underlying diseases. These underlying diseases and gender did not significantly affect the skin microbiome (Supplementary Figures 2, 3; PERMANOVA for gender, unweighted UniFrac: *P* = 0.14; weighted UniFrac, *P* = 0.79). Six bedridden patients used antibacterial drugs; however, there were no significant differences in skin microbiome between the users and non-users of antibacterial drugs (PERMANOVA; unweighted UniFrac, *P* = 0.27; weighted UniFrac, *P* = 0.84), and therefore, all participants were included for further analysis. Four bedridden patients developed PI during observation.

**Table 1:** Participants' characteristics.

**Characteristics**	**Healthy young (*n* = 19)**	**Ambulatory older people (*n* = 18)**	**Bedridden older patients (*n* = 31)**	***P*-value**
Age (years), median (IQR)	22 (21-22)	78 (76.25–83.25)	85 (81–92)	<0.01^a^
Female, *n* (%)	7 (36.8)	11 (61.1)	22 (71.0)	0.08^b^
Braden Scale score, median (IQR)	23 (23-23)	23 (23-23)	12 (11-14)	<0.01^a^
Gastric fistula, *n* (%)	0 (0)	0 (0)	8 (25.8)	<0.01^b^
Onset of PI, *n* (%)	0 (0)	0 (0)	4 (12.9)	0.19^b^
Depth of PI, *n*	–	–	d1:3, d2:1	
**Underlying diseases**, ***n*** **(%)**				
Cerebrovascular disease	0 (0)	0 (0)	18 (58.1)	<0.01^b^
Congestive heart failure	0 (0)	0 (0)	6 (19.4)	0.03^b^
Dementia	0 (0)	0 (0)	3 (9.7)	0.24^b^
Diabetes mellitus	0 (0)	1 (5.6)	3 (9.7)	0.47^b^
Urinary tract infection	0 (0)	0 (0)	3 (9.7)	0.24^b^
**Medications**, ***n*** **(%)**				
Steroid drug	0 (0)	0 (0)	1 (3.2)	1.00^b^
Antibacterial drug	0 (0)	0 (0)	6 (19.4)	0.02^b^
Live bacterial drug	0 (0)	1 (5.6)	5 (16.1)	0.12^b^

a *Kruskal–Wallis test*.

b *Fisher's exact test*.

### Difference in Skin Microbiome Composition Between Bedridden and Healthy Participants

**Figure 1:**
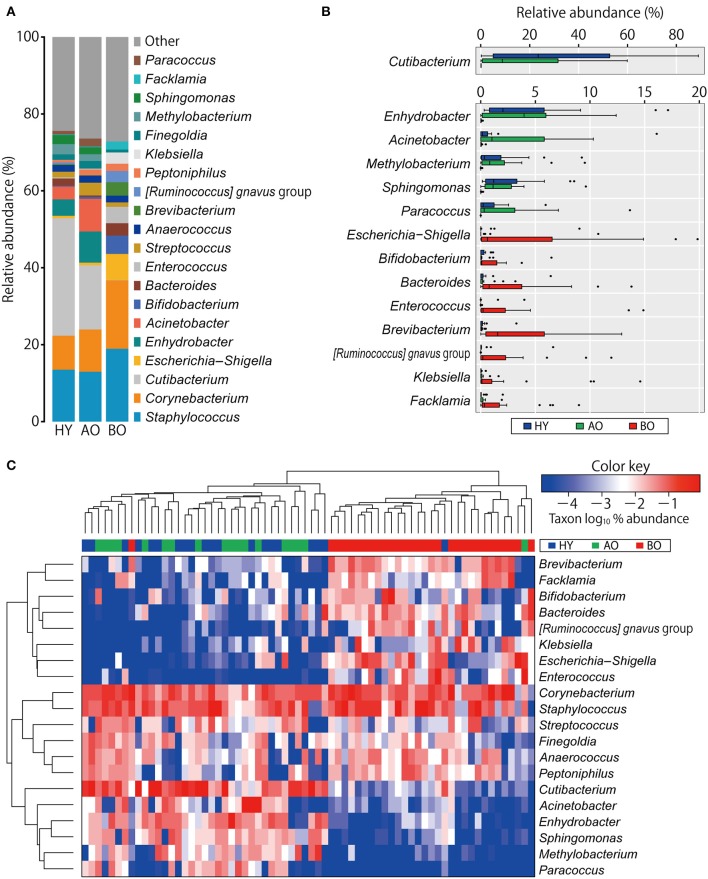
Comparison of bacterial relative abundance. **(A)** Relative abundance of top 20 genera. **(B)** Genera with significant differences among the groups (refer to Supplementary Table 1 for the actual *P*-values). The heatmap shows log10-transformed relative abundance of genera, with dendrograms created using Manhattan distance and the unweighted pair group method with arithmetic mean method **(C)**. Colors above the heatmap indicate the three participant groups: blue for healthy young (HY), green for ambulatory older people (AO), and red for bedridden older patients (BO).

Figure 1A shows compositions of the skin microbiome. The relative abundance of some genera showed significant differences among the three groups (Figure 1B and Supplementary Table 1). Relative abundances of *Cutibacterium* spp. and *Enhydrobacter* spp. were significantly lower in bedridden participants (Figure 1B and Supplementary Table 1). In contrast, relative abundances of *Escherichia–Shigella* spp., *Bifidobacterium* spp., *Bacteroides* spp., *Enterococcus* spp., *Brevibacterium* spp., *Klebsiella* spp., etc. were significantly higher in the bedridden older patient group than in the healthy young and ambulatory older people groups (Figure 1B and Supplementary Table 1). We visualized further the bacterial composition of all participants with a heatmap and dendrograms. There were two apparent clusters in an individual manner (healthy young- and ambulant older people-dominant cluster vs. bedridden older patient-dominant cluster) and three clusters in a bacterial taxonomic manner (bedridden older patients-prone, balanced, and healthy young and ambulant older people-prone) (Figure 1C). The detailed microbial profiles for all participants were as in Supplementary Figure 4.

### Beta Diversity

**Figure 2:**
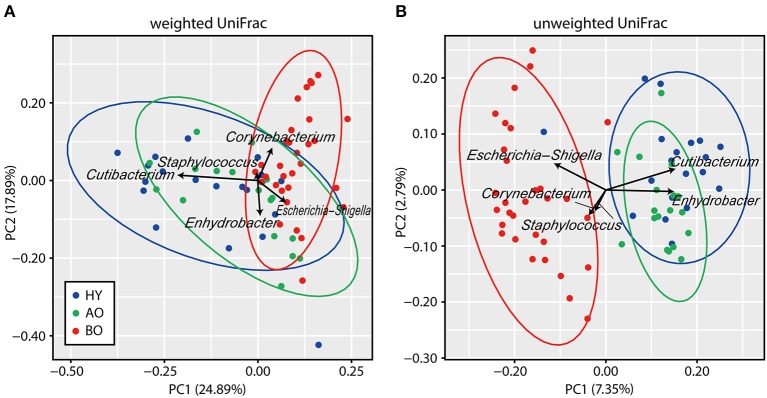
Results of beta diversity analyses. **(A,B)** Biplots based on weighted UniFrac **(A)** and unweighted UniFrac **(B)** are shown. Each color represents a participant group: blue for healthy young (HY), green for ambulatory older people (AO), and red for bedridden older patients (BO). Ellipses represent 95% confidence region of each group. Arrows represent the projection of each genus on the principal coordinate analysis. PC, principal coordinate.

PCoA analysis of beta diversity showed that bedridden participants formed apparently different clusters from healthy young and ambulatory older people groups (Figure 2; PERMANOVA: weighted UniFrac, *P* < 0.01; unweighted UniFrac, *P* < 0.01). Biplot revealed that bedridden older patients were prone to harbor more *Escherichia–Shigella* spp., *Corynebacterium* spp., and *Staphylococcus* spp., and less *Cutibacterium* spp. and *Enhydrobacter* spp.

### Alpha Diversity

**Figure 3:**
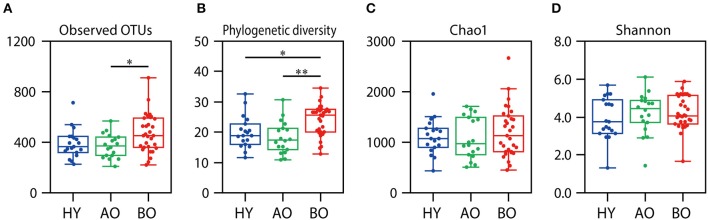
Results of alpha diversity analyses. Rarefaction analyses of the number of observed operational taxonomic units (OTUs) **(A)**, phylogenetic diversity **(B)**, Chao1 **(C)**, and Shannon index **(D)** are shown. Box plots show each metric value at 13,783 depth. *P < 0.05 and **P < 0.01 in Steel–Dwass test compared to each other.

The value of observed OTUs and phylogenetic diversity were significantly higher in bedridden patients than in healthy young people and ambulatory older people (Figure 3A, observed OTUs: *P* = 0.032 between ambulatory and bedridden; Figure 3B, phylogenetic diversity: *P* = 0.026 between young and bedridden, *P* < 0.01 between ambulatory and bedridden). Shannon index and Chao1 showed no significant differences among all groups (Figures 3C,D; *P* = 0.38 in Shannon index, *P* = 0.85 in Chao1 by Kruskal–Wallis test). These comparisons were made after all sequences had been evenly rarefied at 13,783 depth (minimum read number among all samples).

### Relationship Between Skin Physiological Functions and Skin Microbiome

**Figure 4:**
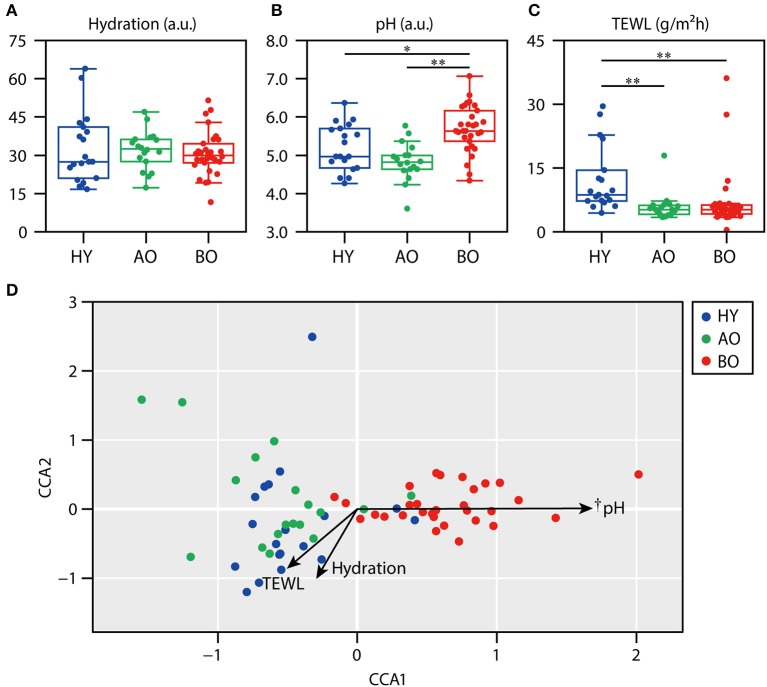
Relationship between skin physiological functions and skin microbiome. The results of skin hydration **(A)**, skin pH **(B)**, and transepidermal water loss (TEWL) **(C)** are shown. **(D)** The result of canonical correspondence analysis (CCA). *P < 0.05 and **P < 0.01 in Steel–Dwass test compared to each other. ^†^*P* < 0.01 in the significance of constraints.

The data of skin physiological functions are shown in Figures 4A–C. As the levels of sebum were too low (only 0 or 1 in the readout) to serve reliable information (data not shown), we excluded the data of sebum level from further analysis. The data of bedridden patient ID 26 was not included in further analysis because of the refusal of the measurements. Skin hydration showed no significant difference among all groups (Figure 4A; *P* = 0.72 by Kruskal–Wallis test). Skin pH (Figure 4B) was significantly higher in bedridden patients than in healthy young (*P* = 0.043) and ambulatory older people (*P* < 0.01). TEWL (Figure 4C) was significantly lower in ambulatory older people (*P* < 0.01) and bedridden patients (*P* < 0.01) than in healthy young people.

Next, we examined the relationship between skin physiological functions and skin microbiome using CCA. The CCA plot showed that the plots of bedridden participants were significantly placed along with the increase in skin pH (Figure 4D; *P* < 0.01). These results show that skin microbiome of bedridden older patients seems to be related to skin pH. The correlations between the relative abundance of each species and skin physiological functions are summarized in Supplementary Table 2.

### Closeness of Skin and Wound Microbiome After PI Onset

In this study, four bedridden participants (12.9%) developed PIs during the observation period (2 months). The positions of PI onset were sacrum (ID 27, depth = d1), coccyx (ID 20, d2; ID 23, d2), and right greater trochanter (ID 28, d1) (Supplementary Figure 1).

**Figure 5:**
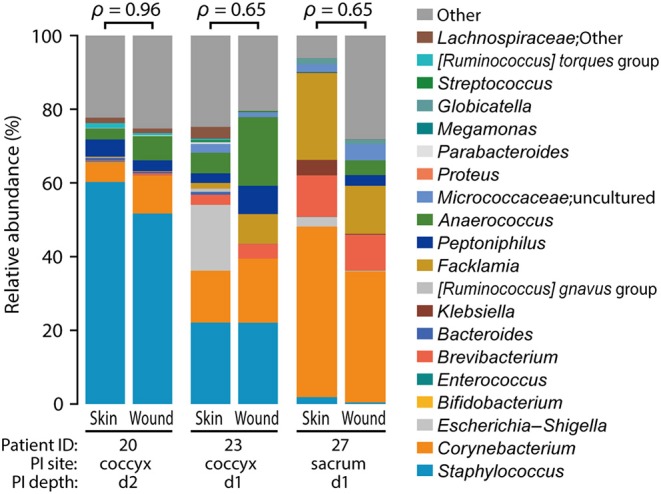
Similarity between before and after pressure injury (PI) onset. Spearman's correlation coefficients (ρ) are shown above the bar graphs. The positions of PI onset were coccyx (ID: 20 and 23; d2 and d1) and sacrum (ID 27; d1) (refer to Supplementary Figure 1).

We examined the closeness of the skin microbiome at the baseline and that after the onset of PI in each participant. As one participant (ID 28) had a PI away from the measurement site (Supplementary Figure 1; right greater trochanter), ID 28 was excluded from this analysis. The compositions of skin microbiome at baseline and after PI onset were similar (Figure 5; Spearman's correlation coefficients ρ = 0.65–0.96).

### Differences in Patients' Information, Skin Microbiome, and Skin Physiological Functions Between PI Onset and Non-onset Groups Among Bedridden Patients

Supplementary Table 3 shows the information of the PI-onset and non-onset group among bedridden patients; there were no significant differences in patients' characteristics between the two groups. In addition, between the PI-onset and non-onset groups, there were no significant differences in skin microbiome at the baseline (Supplementary Figure 5A), no apparent clusters in beta diversities (Supplementary Figures 5B,C; PERMANOVA; weighted UniFrac, *P* = 0.96; unweighted UniFrac, *P* = 0.87), and no significant differences in skin physiological functions, peak interface pressure, or Braden scale total score (Supplementary Figure 6).

## Discussion

In this study, we analyzed the skin microbiomes of bedridden older patients and compared them with that of healthy participants. Consequently, we could show that the skin microbiome of bedridden older patients was clearly distinguishable from that of young or age-matched ambulatory individuals. We surveyed bedridden patients longitudinally until the onset of PI. We could show the remarkable closeness of skin and wound microbiome, implying that the risk of wound infection might be related to intact skin microbiome just before the onset of PIs.

### Skin Dysbiosis in Bedridden Older Patients

The relative abundance of *Cutibacterium* spp. was significantly higher in the young than in the older participants (Figure 1). This finding was in line with the studies that showed reduced *Cutibacterium* spp. abundance with aging (7, 39). We found it interesting that the relative abundance of *Cutibacterium* spp. in bedridden patients was significantly lower than that of ambulatory older people (Figure 1B; 0.1 vs. 16.7%; *P* < 0.01), although these two groups were similar in age (Table 1; median, 85 vs. 78 years old; *P* = 0.06). *Cutibacterium* spp. are known to grow in sebum-rich conditions by metabolizing triglycerides into free fatty acids for their colonization (40, 41). The bedridden condition has been reported to increase the risk of xerosis (i.e., pathological skin dryness), where sebum is depleted (42). Older people are reported to have lower sebum production (43, 44). It is possible, therefore, that the lower levels of *Cutibacterium* spp. can be attributed to the decreased level of sebum in elderly patients, although we could not prove the reduction of sebum in the bedridden patients, presumably due to the low sensitivity of the equipment. In addition, *Enhydrobacter* spp., which have been observed as part of the normal skin flora (45), were detected at significantly lower levels in bedridden patients (Figure 1B).

On the other hand, the number of observed OTUs, phylogenetic diversity indices, and relative abundance of *Escherichia*–*Shigella* spp., *Bifidobacterium* spp., and *Enterococcus* spp. of the bedridden older patients were significantly higher than those of healthy young individuals and ambulant older people (Figures 1, **3**). These bacteria are commonly found in the gut or feces (46, 47), but less often on the skin (1). This could be explained by the fact that all the bedridden participants used diapers. The use of diapers increases the risk of feces attachment to the sacral and coccyx sites (48), which can cause contamination of fecal bacteria on the skin. In this study, however, we collected the skin microbiome after cleansing the area with sterile normal saline, and the collection method (tape stripping) has been found to target a deeper site of the skin than the swabbing method, which can target the superficial bacteria (26). In addition, the skin microbiome in the target site did not significantly change after 2 months (Supplementary Figure 7; PERMANOVA; weighted UniFrac, *P* = 0.19; unweighted UniFrac, *P* = 0.15). Therefore, we assume that the higher abundance of gut-related bacteria detected on the bedridden patients' skin cannot be fully explained merely by contamination but that a reasonable portion of such bacteria may be colonized inside the skin and persist for a longer time. The microenvironment around the skin under the diaper may account for the increase in anaerobes (Supplementary Figure 8; *P* = 0.02 in healthy young participants vs. bedridden older patients), as the use of diapers can create occlusive conditions, making the partial oxygen pressure lower (48). Incidentally, the back-skin's microbiome in the bedridden older patients was also significantly different from healthy participants, with a high abundance of gut-related bacteria *Klebsiella* spp. and *Enterococcus* spp. (Supplementary Figures 9, 10). Roghmann et al. have revealed similar findings (less commensals and more feces-related *Proteus* spp., *Escherichia coli*, and *Enterococcus* spp. on the femoral skin) in the ambulatory or wheelchair-aided older people of nursing homes (9). Although the study did not mention the use of diapers, it can be speculated that the gut-related bacteria on the skin of hospitalized older patients increases even in the regions where the diaper is not directly attached.

In sum, the bedridden patients' skin showed dysbiotic conditions in terms of less commensals (e.g., *Cutibacterium* spp. and *Enhydrobacter* spp.) and more gut-related bacteria (e.g., *Escherichia–Shigella* spp., *Bifidobacterium* spp., and *Enterococcus* spp.).

### Differences in Skin Physiological Functions of Bedridden Older Patients

Next, we showed that the sacral skin pH was significantly higher in the bedridden older patients compared to the other groups (Figure 4B). The high skin pH of bedridden patients can be explained by the decreased relative abundance of *Cutibacterium* spp. *C. acnes* decomposes sebum and produces fatty acids, which keeps the skin pH mildly acidic (2). In this study, skin pH and relative abundance of *Cutibacterium* spp. indeed showed a negative correlation (Supplementary Table 2; ρ = −0.434, *P* < 0.01). Another possibility is, as described above, the sustained contact with urine and/or feces under a diaper. Skin commensals and urease in feces can break down the urea in urine into ammonia, which can cause an increase in skin pH (49). Considering that *C. acnes* prefers slightly more alkaline conditions to grow (pH 6.0–7.0) (50) than the normal skin (pH ~5) (11), and the sacral skin pH of the bedridden older patients was within that alkaline range (Figure 4B), the mechanism in which the high pH suppresses the growth of *C. acnes* can be considered unlikely.

Importantly, a higher skin pH may allow easy colonization of transient bacteria to the skin (2, 11). In fact, this study showed the significant increase in gut-related bacteria in the bedridden participants (Figure 1), and the skin pH and relative abundance of *Escherichia*–*Shigella* spp., *Enterococcus* spp., and *Klebsiella* spp. showed significant positive correlations (Supplementary Table 2). These bacteria are notoriously known to exhibit drug resistance and sometimes cause fatal nosocomial infections (51–54). These findings suggest the importance of maintaining the skin physiological conditions better, not only in terms of skin hygiene but also in terms of restoring skin physiological functions to a healthy state to prevent infections in hospitals.

We also observed a significant decrease in TEWL in the older individuals (Figure 4C), which was in line with previous findings (55, 56). TEWL reflects the rate of water evaporation from inside the skin and thus has been used as an index of skin barrier function. Although lower TEWL is considered to indicate higher skin barrier function (57), TEWL can be altered not only by the skin barrier function but also by the levels of skin hydration, sebum, thickness of stratum corneum cells, and other unknown factors (58); thus, it does not necessarily reflect the actual permeability of the skin (57). The interpretation of lower TEWL in the bedridden older patients is, therefore, inconclusive.

Taken together, bedridden older patients tended to show higher skin pH and lower TEWL in association with a decrease in commensals and an increase in pathogenic bacteria.

### Relationship Between Skin and Wound Microbiome Before and After PI Onset

We found similarities in wound and skin microbiome within the same patients after PI onset, except one whose wound was on the right greater trochanter (Figure 5). Additionally, the wound microbiome, as well as the skin microbiome, showed higher abundance of gut-related bacteria (Figure 5). These findings suggest that wound microbiomes with a high abundance of pathogens are formed from the adjacent skin microbiome, and the bacteria on the skin can remain and persist on the wound, which may ultimately cause wound infection. In previous studies, *Corynebacterium* spp., *Enterococcus faecalis, Bacteroides fragilis*, and *Peptostreptococcus* spp. were isolated from severe necrotizing wounds developed from PIs (59), and the PI was considered a “reservoir” of multidrug resistant *Enterobacteriaceae* and Gram-negative bacilli (60); bacteria of these genera were also observed at the wound site in this study. Infected PIs are reported to pose a risk of bacteremia-related death with an odds ratio of 29.95 (61).

Given that the wound microbiome can be formed by the bacteria on the surrounding skin (19, 20), we underscore the importance of more stringent cleansing of the skin before PI onset in a daily routine to reduce the chance of wound infection by pathogenic bacteria that persist on the skin. As the skin pH in the bedridden older patients was significantly higher than other participants (Figure 4), and the higher skin pH may deteriorate the skin barrier function [for review, see (62)], mildly acidic detergents and/or skincare products are recommended for cleaning up the skin of older patients.

### Relationship Between PI Onset and Skin Parameters

Interestingly, not only skin microbiome and skin physiological functions but also any PI-related parameters, including age, peak interface pressure and Braden Scale score, failed to predict the onset of PI (Supplementary Table 3 and Supplementary Figures 5, 6). The Braden Scale has been widely used as an assessment tool for PI onset and prevention (63); however, the scale alone has been shown to have moderate predictive validity and thus cannot very exactly predict the onset of PIs (64–66). Interface pressure is one of the most important factors in the onset of PIs, and reducing the pressure takes a priority for PI prevention (67). Both in the PI-onset and non-onset groups, the median of the peak interface pressure at the sacral region exceeded the threshold of 32 or 40 mmHg (68) but were not significantly different in relation to PI onset [Supplementary Table 3 and Supplementary Figure 6D; 47.2 (IQR, 28.0–58.3) vs. 43.55 (IQR, 34.25–53.95) in non-onset vs. PI-onset groups; *P* = 0.73]. These results imply the involvement of other factors in the onset of PIs in bedridden patients, which poses the necessity for further study.

### Limitations

We should acknowledge several limitations of this study. First, we have limited the measurement area to the sacral region because we first focused on the relationship between PI and skin parameters such as skin microbiome and skin physiological functions. As the compositions of skin microbiome greatly differ depending on the area (1) and the same is true for older patients (9), other parts of the body should be of interest.

Second, although we showed that the distinct skin microbiome was related to higher pH and lower TEWL, we could not show the causal relationship between the two events. As dysbiosis can affect the skin's physiological functions and vice versa, longer observation is required to prove the temporal and causal interactions of skin physiological functions and skin microbiome.

Third, it was not possible to collect data from *bedridden young* patients in comparison with *bedridden older* patients or *healthy older* people, as the hospital in this study is admitting mostly older patients and we could not find any bedridden young patients. To clarify if the dysbiotic condition can be attributed to aging or the bedridden condition, or both, the data from bedridden young patients would be of interest.

Fourth, our cohort size was so small that careful attention would be required to generalize this study. We deem, however, that the conclusion of this study would not be largely compromised due to the small size of the cohort, as the findings of this study were applicable to most of the participants, not only to the specific ones. Nonetheless, we could not rule out the possibility that the unique microbiome of bedridden older patients in this study might be because of the hospital environment and/or regional characteristics, although similar findings are reported in the different setting (9). Larger cohorts with multicenter setting will be required for future researches.

Lastly, as there were no patients who developed wound, skin, and/or soft tissue infection during the observation period, we could not prove whether the difference in skin microbiome in the bedridden older patients was related to skin infection. To determine these factors, a larger and longer cohort observation would be required.

## Conclusion

In this study, we showed that the skin microbiome and skin physiological functions of bedridden older patients were different compared to those of healthy participants. We also showed that the skin microbiome can propagate into the wound among the patients who developed PI. Because the skin of bedridden older patients showed higher abundance of gut-related bacteria, and the wound microbiome was similar to the skin microbiome, skin dysbiosis of bedridden older patients may be a risk factor of wound infection after PI development.

## Data Availability Statement

All the sequence data are available on the DNA Data Bank of Japan (DDBJ; accession number is DRA007616).

## Author Contributions

SN, KO, JS, and SO conceived the study and critically reviewed and edited the manuscript to the final version. SN, KO, TU, KS, EM, KM, MM, YM, MA, DA, and JS collected the samples and performed the experiments. SN, KO, TU, KS, EM, JS, and SO analyzed the data. SN and KO wrote the original draft of the manuscript. JS and SO acquired the funding and supervised the study. All authors approved the final version of the manuscript.

### Conflict of Interest

The authors declare that the research was conducted in the absence of any commercial or financial relationships that could be construed as a potential conflict of interest.
