# Supplementary material for: Distinct Skin Microbiome and Skin Physiological Functions Between Bedridden Older Patients and Healthy People: A Single-Center Study in Japan
Source: Front Med (Lausanne). 2020 Apr 8;7:101. doi: 10.3389/fmed.2020.00101 (PMC7156624; doi:10.3389/fmed.2020.00101)
Supplement: Supplementary file 1 [file Data_Sheet_1.PDF]

Supplementary Material

Distinct skin microbiome and skin physiological functions between bedridden older patients and healthy people: A single-center study in Japan

Satoshi Nagase[1], Kazuhiro Ogai[2*], Tamae Urai[3†], Kana Shibata[1#], Emi Matsubara[1#], Kanae Mukai[4], Miki Matsue[1], Yumiko Mori[1], Miku Aoki[4‡], Defa Arisandi[4], Junko Sugama[2,3,4], Shigefumi Okamoto[1,2*]

[*]Correspondence:
Kazuhiro Ogai: kazuhiro@staff.kanazawa-u.ac.jp
Shigefumi Okamoto: sokamoto@mhs.mp.kanazawa-u.ac.jp

1. Supplementary Figures and Tables

1.1 Supplementary Figures

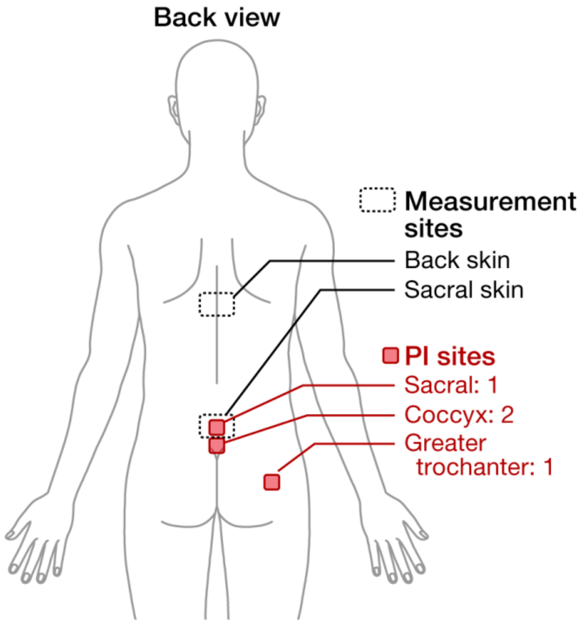

Supplementary Figure 1. Positions of the measurement area and the onset of pressure injury (PI).

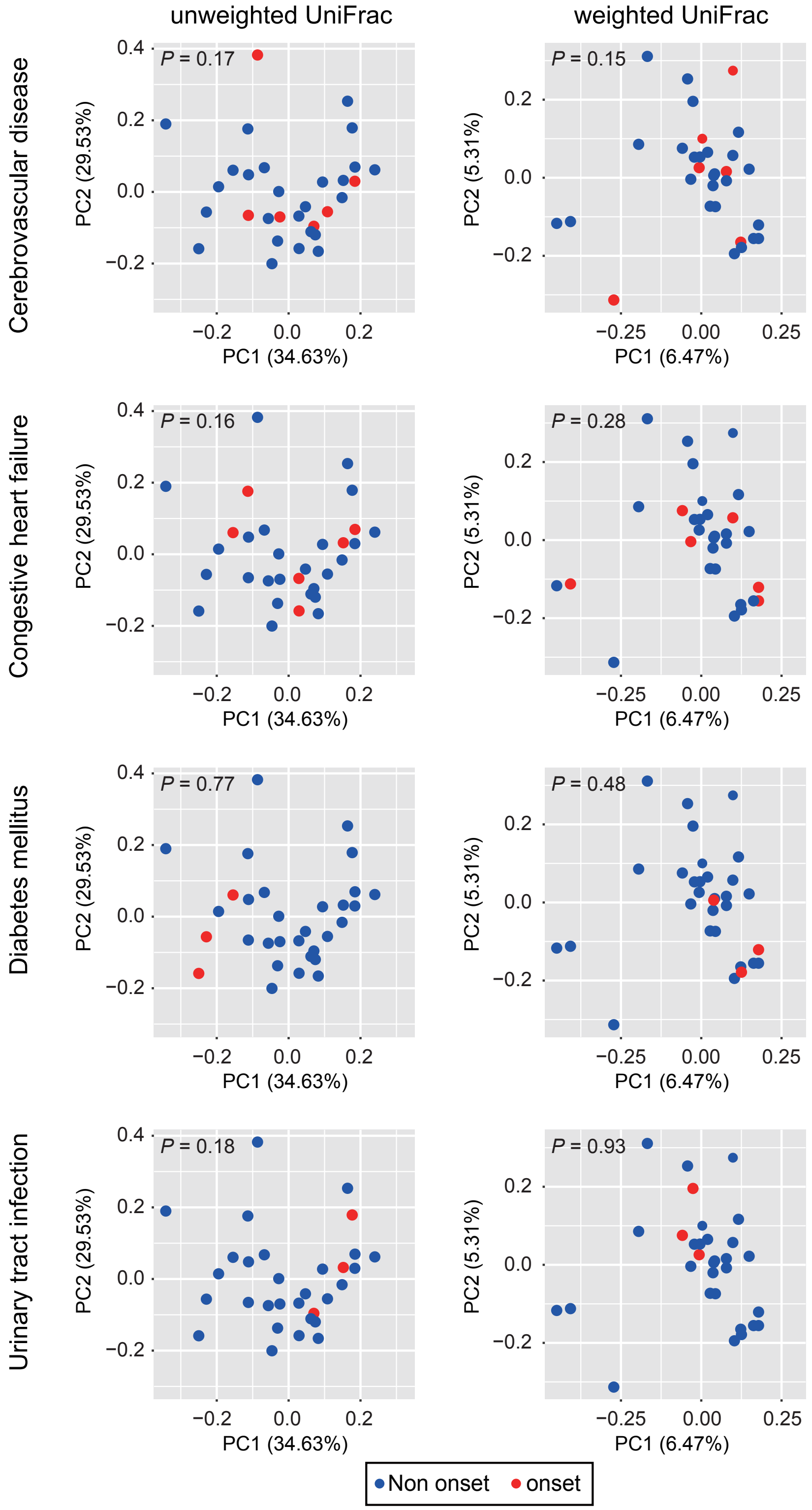

Supplementary Figure 2. Relationship between underlying diseases and skin microbiome. The principal coordinates of the beta diversity (weighted and unweighted UniFrac) indices were stratified by the onset of each underlying disease. *P*-values were calculated for the permutational analysis of variance test. Red points and blue points denote the onset group and the non-onset group, respectively. PC, principal coordinate.

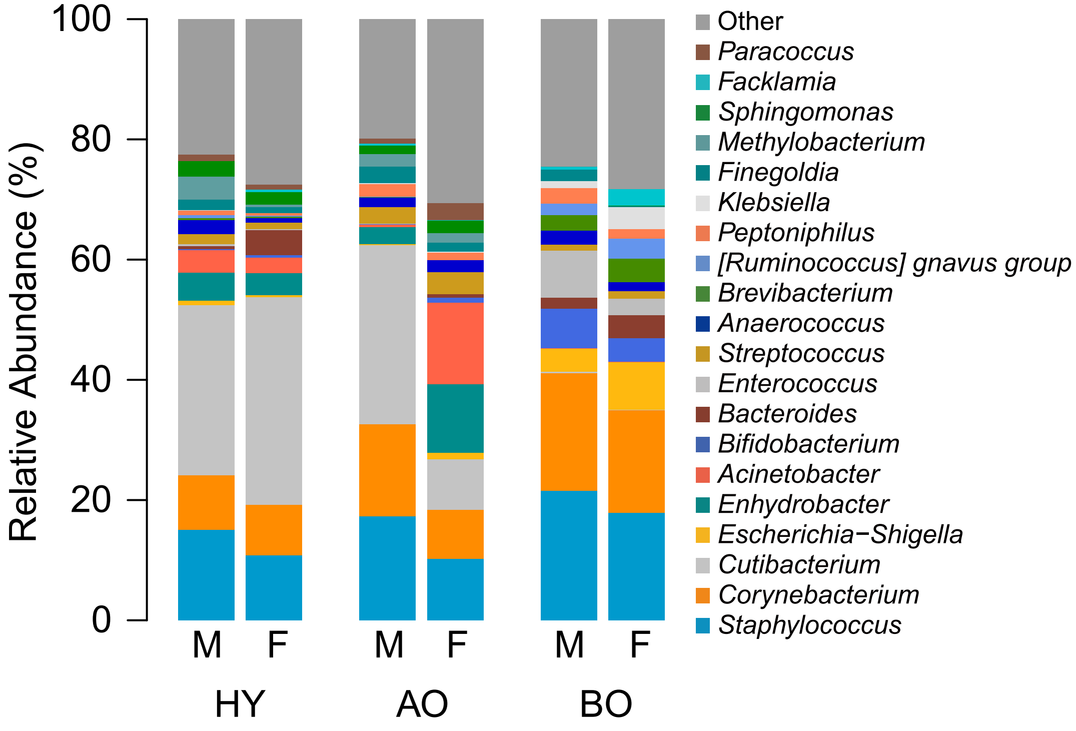

Supplementary Figure 3. Relative abundances of top 20 genera among groups between male and female. HY, healthy young; AO, ambulatory older people; BO, bedridden older patients; M, male; F, female.

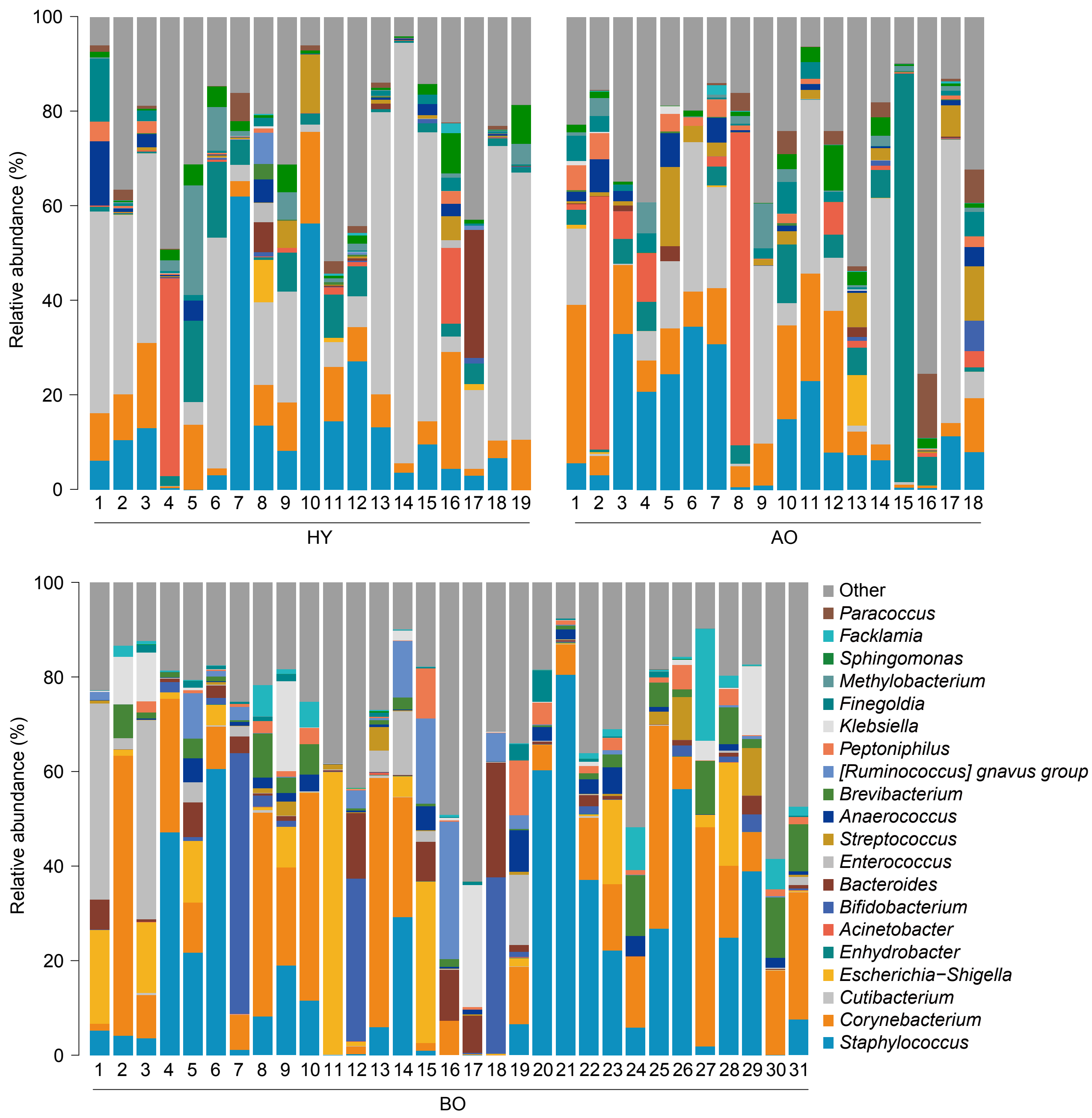

Supplementary Figure 4. Relative abundances of top 20 genera for each participant. HY, healthy young; AO, ambulatory older people; BO, bedridden older patients.

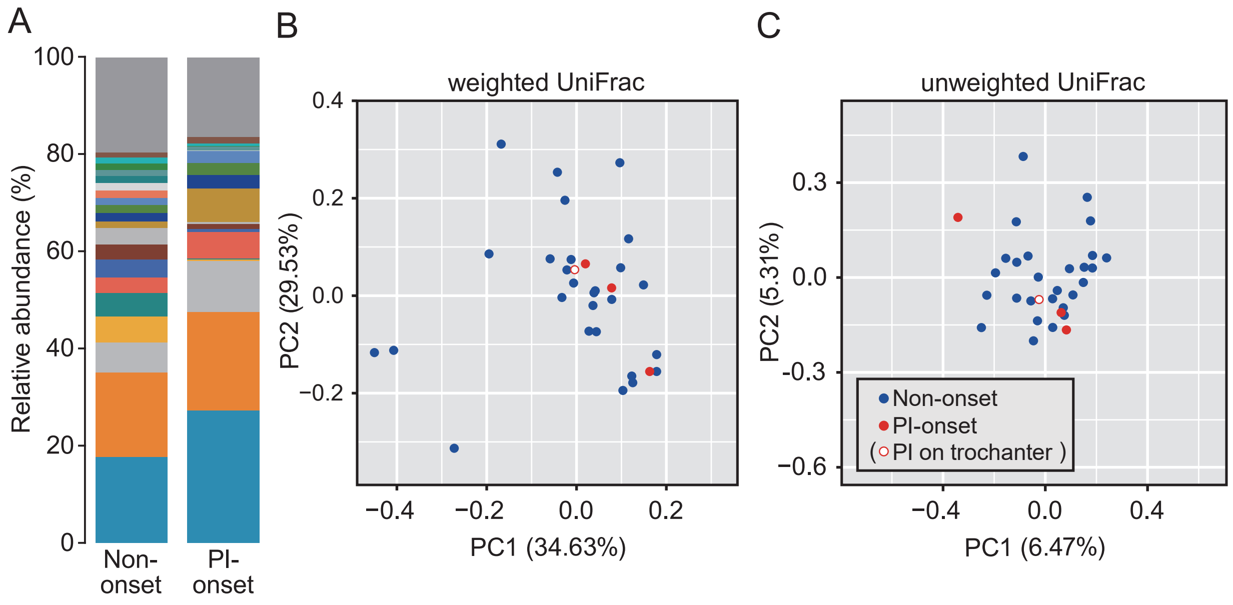

Supplementary Figure 5. Relationship between PI onset and skin microbiome. (A) Relative abundance of top 20 genera for PI-onset group and non-onset group. (B, C) Beta diversity analysis between PI-onset and non-onset groups, based on weighted UniFrac (B) and unweighted UniFrac (C). Red denotes PI-onset group and blue denotes non-onset group. An open circle (○) plot in each panel indicates the individual who developed PI on the right greater trochanter (refer to Supplementary Figure 1). PC, principal coordinate.

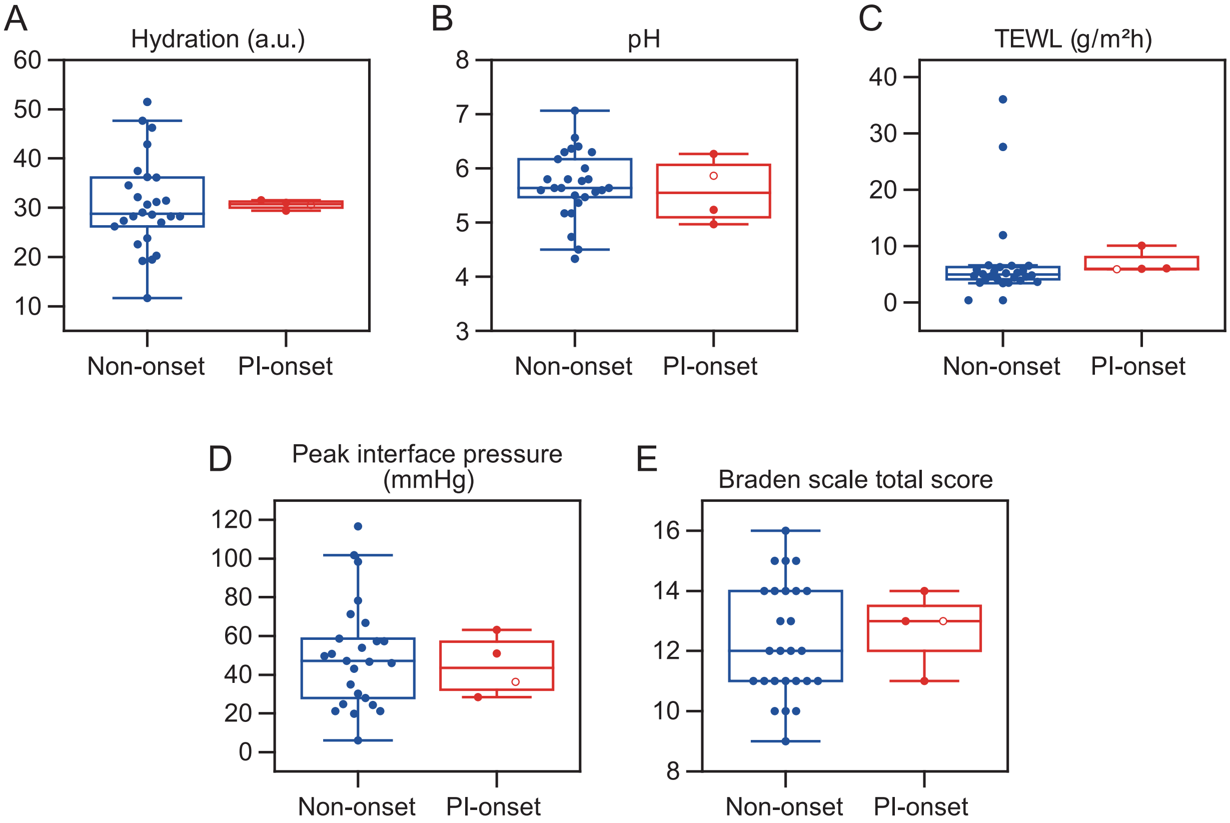

Supplementary Figure 6. Measurement results of skin hydration (A), skin pH (B), transepidermal water loss (TEWL) (C), peak interface pressure (D), and Braden Scale total score (E) in PI-onset and non-onset groups. An open circle (○) plot in each panel indicates the individual who developed PI on the right greater trochanter (refer to Supplementary Figure 1).

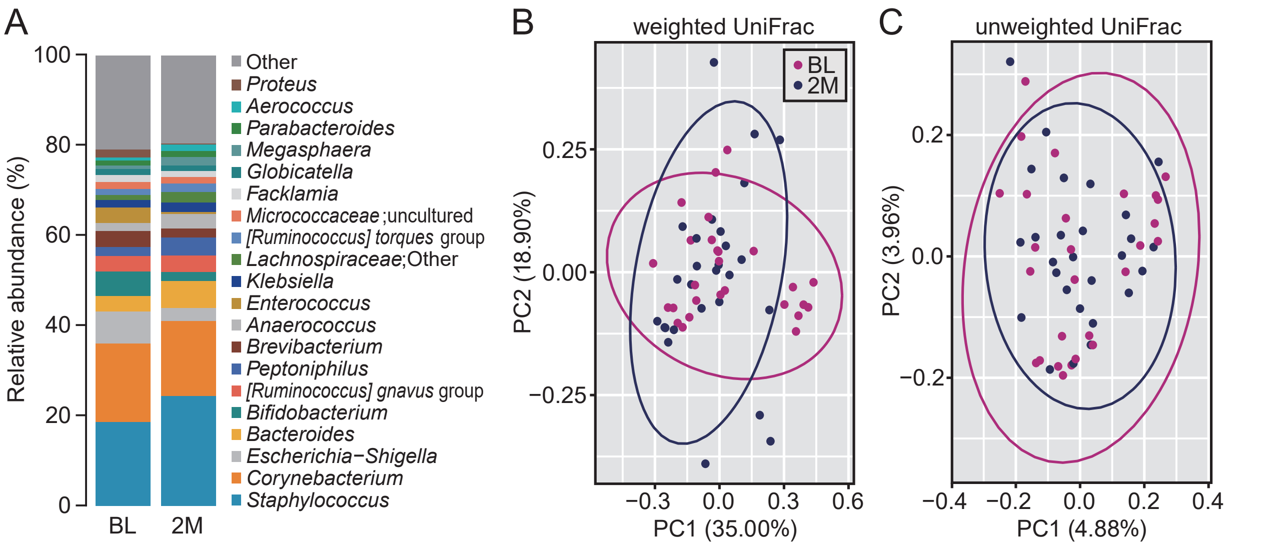

Supplementary Figure 7. Microbial changes in 2 months. (A) Relative abundance of top 20 genera at baseline (BL) and at 2 months (2M). (B, C) Beta diversity analysis based on weighted UniFrac (B) and unweighted UniFrac (C) at baseline (BL; navy blue) and 2 months later (2M; pink). Ellipses represent 95% confidence region of each time point.

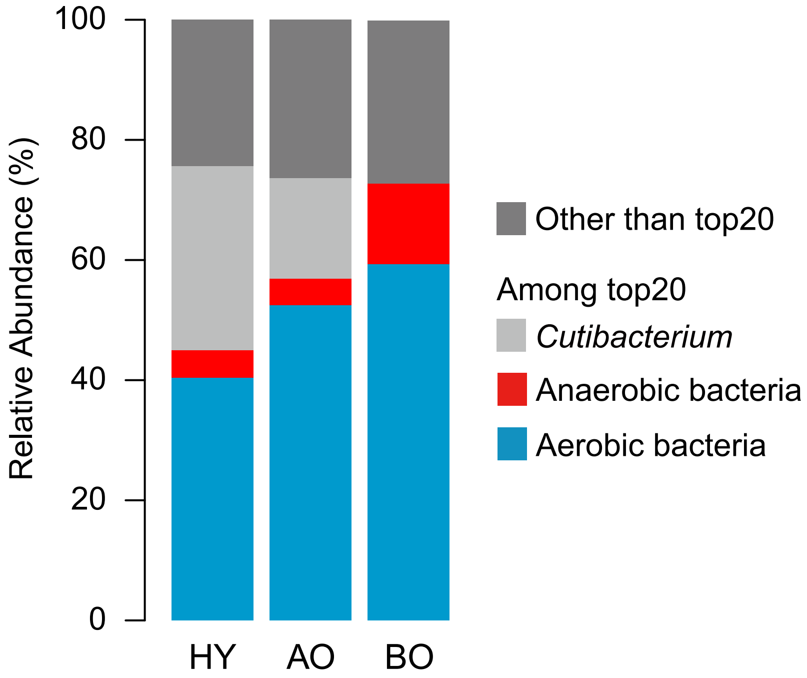

Supplementary Figure 8. Relative abundances of anaerobic and aerobic bacteria among groups on the sacral region (refer to Supplementary Figure 1). HY, healthy young; AO, ambulatory older people; BO, bedridden older patients.

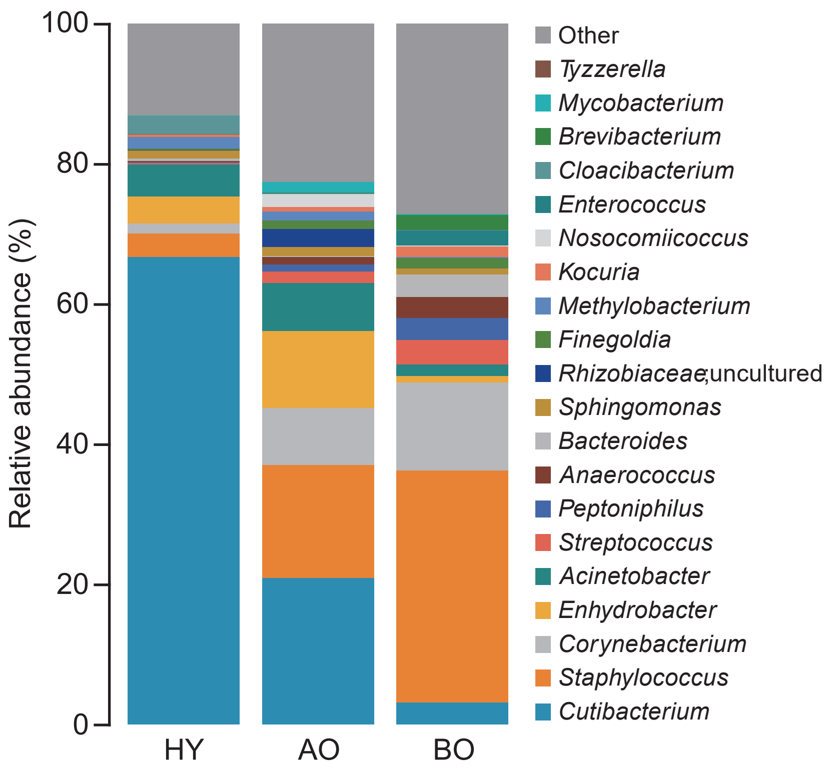

Supplementary Figure 9. Relative abundance of top 20 genera among groups on the back region (refer to Supplementary Figure 1). HY, healthy young; AO, ambulatory older people; BO, bedridden older patients.

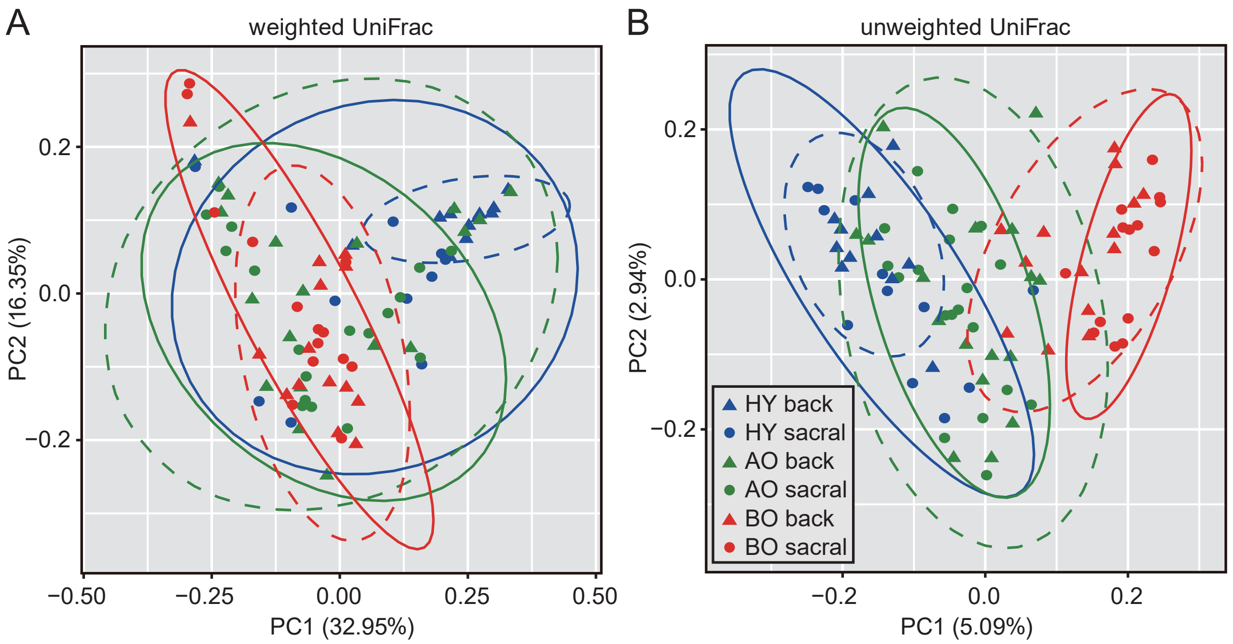

Supplementary Figure 10. Comparison of microbiome between the back region and sacral region. Beta diversity analysis based on weighted UniFrac (A) and unweighted UniFrac (B). Each color of the plot represents each participant group: blue for healthy young (HY), green for ambulatory older people (AO), and red for bedridden older patients (BO). The circle (●) plots are from the sacral region, whereas the triangle (▲) plots are from the back skin. Ellipses represent 95% confidence region of each group; solid lines for the sacral region and dashed line for the back region.

1.2 Supplementary Tables

Supplementary Table 1. *P*-values derived from the Steel–Dwass test for the comparison of relative abundances in three participant groups: healthy young (HY), ambulatory older people (AO), and bedridden older patients (BO) (related to **Figure 1B**).

Supplementary Table 2. Spearman's correlation coefficients between skin physiological function and relative abundance of each genus.

Supplementary Table 3. Patients' characteristics between PI-onset and non-onset groups.
