# Supplementary material for: Distinct Skin Microbiome and Skin Physiological Functions Between Bedridden Older Patients and Healthy People: A Single-Center Study in Japan
Source: Front Med (Lausanne). 2020 Apr 8;7:101. doi: 10.3389/fmed.2020.00101 (PMC7156624; doi:10.3389/fmed.2020.00101)
Supplement: Supplementary file 2 [file Table_1.docx]

**Supplementary Table 1. Adjusted *P*-values of Steel–Dwass test among the three groups for each genus.**

| **Genus** | **HY vs AO** | **HY vs BO** | **AO vs BO** |
| --- | --- | --- | --- |
| *Cutibacterium* | 0.16 | <0.01 | <0.01 |
| *Enhydrobacter* | 0.98 | <0.01 | <0.01 |
| *Acinetobacter* | 0.23 | 0.02 | 0.02 |
| *Methylobacterium* | 0.89 | <0.01 | <0.01 |
| *Sphingomonas* | 0.85 | <0.01 | <0.01 |
| *Paracoccus* | 0.95 | <0.01 | <0.01 |
| *Escherichia*–*Shigella* | 0.96 | <0.01 | <0.01 |
| *Bifidobacterium* | 0.80 | 0.05 | 0.01 |
| *Bacteroides* | 0.68 | <0.01 | <0.01 |
| *Enterococcus* | 0.88 | <0.01 | 0.02 |
| *Brevibacterium* | 0.96 | <0.01 | 0.03 |
| *[Ruminococcus] gnavus* group | 0.10 | 0.03 | 0.04 |
| *Klebsiella* | 0.82 | <0.01 | <0.01 |
| *Facklamia* | 0.75 | <0.01 | <0.01 |

HY, healthy young people; AO, ambulatory older people; BO, bedridden older patients.

Please refer to Figure 1B.
