# Supplementary material for: Distinct Skin Microbiome and Skin Physiological Functions Between Bedridden Older Patients and Healthy People: A Single-Center Study in Japan
Source: Front Med (Lausanne). 2020 Apr 8;7:101. doi: 10.3389/fmed.2020.00101 (PMC7156624; doi:10.3389/fmed.2020.00101)
Supplement: Supplementary file 3 [file Table_2.docx]

**Supplementary Table 2. Spearman’s correlation coefficient between skin physiological functions and the relative abundance of each genus.**

| **Spearman’s correlation coefficient**  **(Skin physiological function vs relative abundance)** | | | |
| --- | --- | --- | --- |
| **Genus** | **pH** | **hydration** | **TEWL** |
| *Staphylococcus* | -0.262* | 0.212 | 0.157 |
| *Corynebacterium* | 0.071 | 0.012 | -0.103 |
| *Cutibacterium* | -0.434** | 0.072 | 0.671** |
| *Escherichia.Shigella* | 0.423** | -0.116 | -0.371** |
| *Enhydrobacter* | -0.411** | -0.054 | 0.546** |
| *Acinetobacter* | -0.336** | -0.140 | 0.205 |
| *Bifidobacterium* | 0.351** | -0.252* | -0.434** |
| *Bacteroides* | 0.278* | 0.013 | -0.464** |
| *Enterococcus* | 0.486** | -0.098 | -0.491** |
| *Streptococcus* | -0.184 | 0.142 | 0.195 |
| *Anaerococcus* | -0.073 | -0.097 | -0.192 |
| *Brevibacterium* | 0.323** | 0.036 | -0.364** |
| *[Ruminococcus] gnavus* group | 0.335** | -0.163 | -0.487** |
| *Peptoniphilus* | 0.019 | 0.006 | -0.258* |
| *Klebsiella* | 0.408** | 0.035 | -0.300* |
| *Finegoldia* | -0.147 | -0.077 | 0.071 |
| *Methylobacterium* | -0.401** | -0.106 | 0.429** |
| *Sphingomonas* | -0.368** | -0.043 | 0.584** |
| *Facklamia* | 0.359** | -0.020 | -0.330** |
| *Paracoccus* | -0.385** | 0.050 | 0.505** |

TEWL, transepidermal water loss.

**P* < 0.05 and ***P* < 0.01
