# Supplementary material for: Distinct Skin Microbiome and Skin Physiological Functions Between Bedridden Older Patients and Healthy People: A Single-Center Study in Japan
Source: Front Med (Lausanne). 2020 Apr 8;7:101. doi: 10.3389/fmed.2020.00101 (PMC7156624; doi:10.3389/fmed.2020.00101)
Supplement: Supplementary file 4 [file Table_3.docx]

**Supplementary Table 3. Patients’ characteristics between the pressure injury-onset group and non-onset group.**

| **Characteristics** | **Non-onset group**  **(*n* = 27)** | **Onset group**  **(*n* = 4)** | ***P*-value** |
| --- | --- | --- | --- |
| Age (years), median (IQR) | 85 (82.5–92.5) | 84.5 (76–90.5) | 0.60^a^ |
| Female, *n* (%) | 19 (70.4) | 3 (75) | 1.00^b^ |
| Braden scale, median (IQR) | 12 (11–14) | 13 (12.5–13.25) | 0.96^a^ |
| Gastric fistula, *n* (%) | 6 (22.3) | 2 (50) | 0.27^b^ |
| Peak interface pressure (mmHg), median (IQR) | 47.2 (28–58.3) | 43.55 (34.25–53.95) | 0.73^a^ |
| PI depth:*n* |  | d1:2, d2:2 |  |
| Underlying disease, *n* (%) |  |  |  |
| Cerebrovascular disease | 15 (55.6) | 3 (75) | 0.62^b^ |
| Congestive heart failure | 6 (22.3) | 0 (0) | 0.56^b^ |
| Dementia | 2 (7.5) | 1 (25) | 0.34^b^ |
| Diabetes mellitus | 3 (11.2) | 0 (0) | 1.00^b^ |
| Urinary tract infection | 3 (11.2) | 0 (0) | 1.00^b^ |
| Medications, *n* (%) |  |  |  |
| Steroid drug | 1 (3.8) | 0 (0) | 1.00^b^ |
| Antibacterial drug | 6 (22.3) | 1 (25) | 1.00^b^ |
| Live bacterial drug | 5 (18.6) | 0 (0) | 1.00^b^ |

IQR, interquartile range; PI, pressure injury.

^a^ Kruskal–Wallis test, ^b^ Fisher’s exact test.
